# HOLISTIC WELLNESS COACHING INTERVENTION FOR OLDER ADULTS IN RESIDENTIAL COMMUNITIES

**DOI:** 10.1093/geroni/igac059.1165

**Published:** 2022-12-20

**Authors:** Philip Clarke, Matthew Fullen, Jennifer Smith

**Affiliations:** Wake Forest University, Winston-Salem, North Carolina, United States; Virginia Tech, Blacksburg, Virginia, United States; Mather Institute, Evanston, Illinois, United States

## Abstract

Many wellness programs for older adults focus on physical health or specific conditions, such as heart disease prevention or diabetes management. To supplement these efforts, there is a need for holistic wellness programming to enhance well-being in later life. We developed and piloted a novel wellness coaching program for residents of senior living communities to address this need. The theoretical framework of the program is based on a holistic, multidimensional wellness model and self-determination theory. Staff members from eight residential communities were trained on wellness coaching techniques, such as motivational interviewing, active listening, and group facilitation skills, before they led the 9-week resident wellness coaching program. A total of 79 residents, ages 71 to 97 (M = 84.3, SD = 6.5), completed the pilot program. The resident wellness coaching program included a mixture of individual and group coaching sessions. The coaching sessions provided wellness education, social support, and space to share progress on wellness goals. Participants completed surveys at three time points: pre-program, post-program, and 1-month follow-up. Residents reported high satisfaction with the overall program and its components. Repeated measures ANOVAs were conducted to examine changes in wellness over time. Comparison of pre- and post-test scores revealed significant improvements in health satisfaction, physical and psychological quality of life, purpose, loneliness, relatedness, and competence. Some of these changes, such as increases in psychological quality of life and decreases in loneliness, persisted at the one-month follow-up. These findings have implications for the development and implementation of wellness coaching programs with older adults.

